# Genetic Diversity among Ancient Nordic Populations

**DOI:** 10.1371/journal.pone.0011898

**Published:** 2010-07-30

**Authors:** Linea Melchior, Niels Lynnerup, Hans R. Siegismund, Toomas Kivisild, Jørgen Dissing

**Affiliations:** 1 Research Laboratory, Faculty of Health Sciences, Institute of Forensic Medicine, University of Copenhagen, Copenhagen, Denmark; 2 Anthropological Laboratory, Faculty of Health Sciences, Institute of Forensic Medicine, University of Copenhagen, Copenhagen, Denmark; 3 Department of Biology, Faculty of Natural Sciences, University of Copenhagen, Copenhagen, Denmark; 4 Leverhulme Centre for Human Evolutionary Studies, University of Cambridge, Cambridge, United Kingdom; Max Planck Institute for Evolutionary Anthropology, Germany

## Abstract

Using established criteria for work with fossil DNA we have analysed mitochondrial DNA from 92 individuals from 18 locations in Denmark ranging in time from the Mesolithic to the Medieval Age. Unequivocal assignment of mtDNA haplotypes was possible for 56 of the ancient individuals; however, the success rate varied substantially between sites; the highest rates were obtained with untouched, freshly excavated material, whereas heavy handling, archeological preservation and storage for many years influenced the ability to obtain authentic endogenic DNA. While the nucleotide diversity at two locations was similar to that among extant Danes, the diversity at four sites was considerably higher. This supports previous observations for ancient Britons. The overall occurrence of haplogroups did not deviate from extant Scandinavians, however, haplogroup I was significantly more frequent among the ancient Danes (average 13%) than among extant Danes and Scandinavians (∼2.5%) as well as among other ancient population samples reported. Haplogroup I could therefore have been an ancient Southern Scandinavian type “diluted” by later immigration events. Interestingly, the two Neolithic samples (4,200 YBP, Bell Beaker culture) that were typed were haplogroup U4 and U5a, respectively, and the single Bronze Age sample (3,300–3,500 YBP) was haplogroup U4. These two haplogroups have been associated with the Mesolithic populations of Central and Northern Europe. Therefore, at least for Southern Scandinavia, our findings do not support a possible replacement of a haplogroup U dominated hunter-gatherer population by a more haplogroup diverse Neolithic Culture.

## Introduction

The oldest human skeletal material present in Southern Scandinavia relates to Mesolithic hunter-gatherers from approx. 7,000 YBP. Around 6,000 YBP the first signs of agriculture appeared, and this gradually changed the society from the band organisation seen among hunter-gatherers to a more complex tribal mode of organisation among the first farmers [1]. During the subsequent Bronze Age (3,800–2,500 YBP) the farming culture expanded rapidly catalysed by a new metal technology, and non-egalitarian ranked societies, so-called chiefdoms, emerged. Southern Scandinavia and the northern part of Europe merged into a larger integral area based on exchange of products from these areas [1]. In the Roman Iron Age (AD 1–400), the relatively small and local political structures were gradually replaced by more extensive political unifications, which were reflected in increasingly hierarchical formations, and there is evidence that Chiefly lineages established alliances also by kinship [2]. The first contacts with the Roman civilisation were established in the Iron Age. True state formation in the Danish territory may first have appeared in the late Viking Age/Early Medieval Age [3]. Especially the Viking Age (AD 750–1050) is characterised by vast expansions in the North Atlantic area, the British Islands and Northern France. Introduction of Christianity around AD 1000 marked the end of the Prehistoric Era in Southern Scandinavia, and the Medieval Period was, as the Viking Age, characterised by contacts with the rest of Scandinavia and Europe based on commerce, pilgrimage and migration [3].

Analysis of extant human DNA has greatly contributed to the understanding of human origins and migrations [4]–[7]. However, an obvious problem when population history is deduced from results with extant human material is that important information regarding population replacements and minor migration events is missed, if genetic information has been lost over long periods of time. This problem may be circumvented if DNA is obtained directly from ancient material when this is available. Genetic analysis of our ancestors may thereby contribute to our understanding of migration patterns, population affinities, replacements, tribal patterns and family structure. However, the retrieval of suitable authentic ancient DNA (aDNA) from ancient human material and the subsequent genetic analysis is time consuming and by no means a trivial task, despite this fact a number of such studies have hitherto been performed [8]–[21]. Cold and/or dry environments are ideal for the long time survival of DNA in the remains of organisms and excellent specimens have been recovered from such climatic locations [9], [22]–[25]. However, most humans live in warmer and more humid environments where the conditions for DNA survival are less ideal, limiting the amount of material suitable for DNA studies.

The University of Copenhagen has a unique collection of approximately 25,000 skeletons from all prehistoric and historic periods of the Danish past. Many of these skeletons are well preserved and retrieval and analysis of DNA may be accomplished. This has allowed us to get a glimpse of our genetic past. Further, thorough archaeological and anthropological data associated with the skeletal material makes it possible to assess the influence of the storage time, preservation and age of the material on the ability to obtain authentic ancient DNA. We have previously presented results from an early Christian cemetery near the former royal town Roskilde [17], from two Roman Iron age settlements near Vordingborg [15] and from a Viking site in Northern Fyn [16]. Here we present results on individuals from 14 additional locations ranging in time from the Mesolithic to the Medieval Age, and the collective material of 56 individuals allows us to assess the genetic diversity in past populations of this part of Southern Scandinavia.

## Materials and Methods

### Archaeological sites and human remains

**Figure 1:**
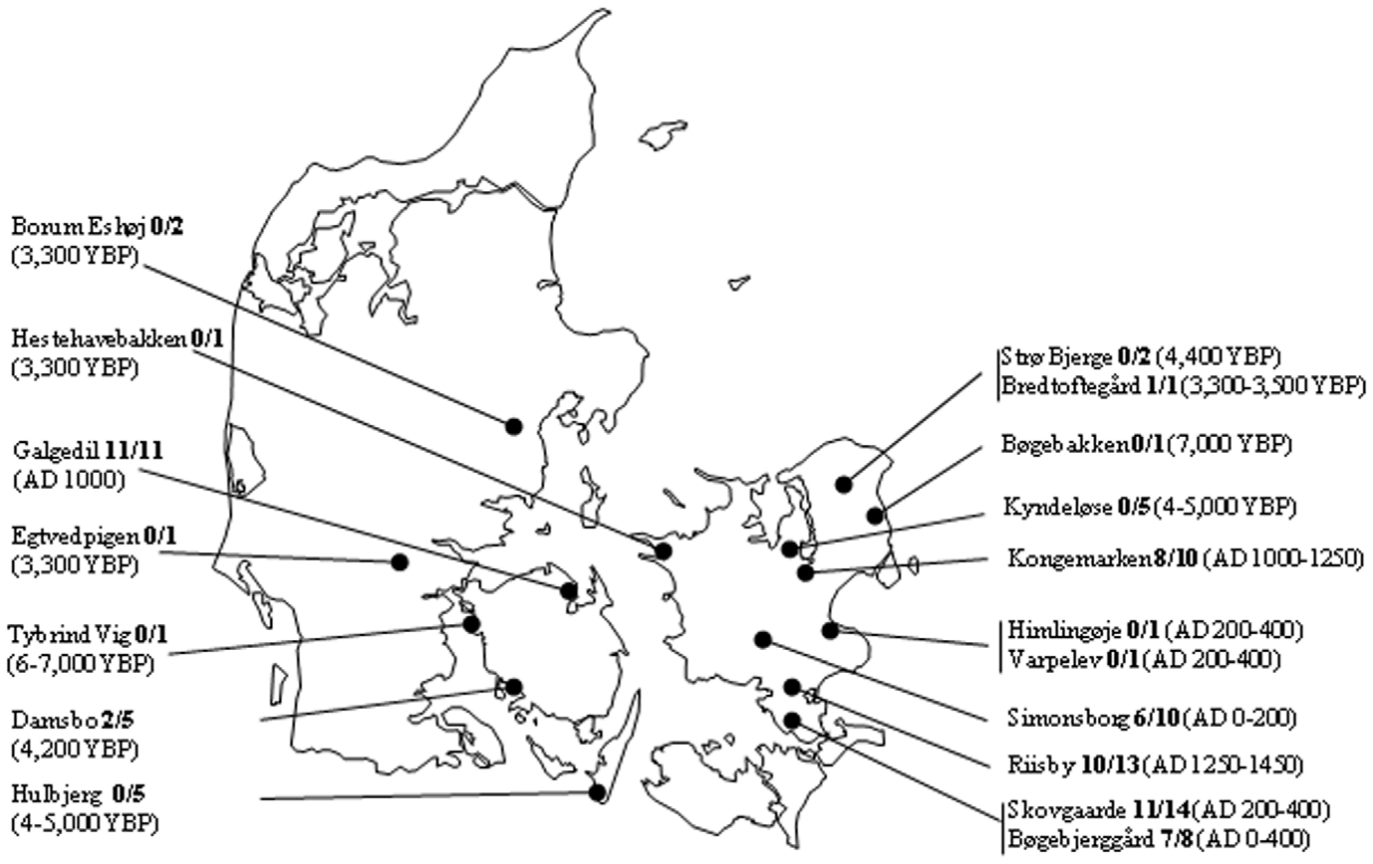
Map of Denmark. Locations and time periods of the sites investigated. The success rates of DNA analyses are shown in bold.

Material (teeth or hair) was sampled from 92 individuals from 18 locations in Denmark as shown in Fig. 1. These samples represented Mesolithic Age (Bøgebakken and Tybrind Vig, ∼6,000–7,000 YBP), Neolithic Age (Strø Bjerge, Damsbo, Kyndeløse and Hulbjerg, ∼4,000–5,000 YBP), Bronze Age (Hestehavebakken, Egtvedpigen, Borum Eshøj and Bredtoftegård, ∼3,300–3,700 YBP), Roman Iron Age (Varpelev, Himlingøje, Skovgaarde, Simonsborg and Bøgebjerggård, ∼AD 1–400), Viking Age (Galgedil, ∼AD 1000), Early Christian period (Kongemarken, AD 1000–1250) and Medieval Age (Riisby, AD 1250–1450). Additional data regarding the archaeological sites can be found in Table S1 and Table S2.

### Ethics Statement

The “Region Hovedstaden” Board of Ethics and Danish Data Protection Agency have approved the usage of DNA from staffs in the present work. Written informed consent was obtained from all staffs involved.

### aDNA work

The methods and materials used and the precautions taken regarding pre-PCR work, chemicals, reagents etc. are described in detail in Melchior et al. (2008) [15], [16]. To ensure the highest possible reliability of the work the most general and widely accepted guidelines for aDNA work were followed [26]–[29]. The following additional elements were applied 1) Miscoding lesions: If no miscoding lesions were identified in at least two of the four overlapping PCR fragments (each containing ∼8 clones), additional PCRs were carried out. If the cloned sequences still lacked any miscoding lesions, the result was rejected as possible contaminants. 2) Staff database: In order to exclude the laboratory staffs, archaeologists and anthropologists as the source of DNA, all individuals involved in the work were mtDNA haplotyped and all aDNA sequences were compared with this database. 3) Control Inuit DNA: To test the overall reliability of the laboratory procedures used and to identify possible contaminants, a tooth from an Inuit skull (Greenland ∼500YBP) was analysed using the standard procedure [15]–[17]. The reason for this additional step is that in contrast to the other research material explored in this study the Inuit sample is not supposed to yield European mtDNA sequences. Thus detection of European or non-Inuit sequences in the Inuit sample would indicate a laboratory based contamination.

### Decontamination procedure

Teeth from the individuals from Damsbo, Bredtoftegård, Strø Bjerge and Tybrind Vig were decontaminated using a modification of the previously published technique, which included wiping of the surface with a cloth soaked with 5% commercial bleach [15], [16]. In the new procedure [30] the tooth was wiped with a dry cloth and powdered pulp was obtained as described in Melchior et al. (2008) [15]. The powder was suspended in 500 µl 2% sodium hypochlorite (Sigma Aldrich, St. Louis, MO) for 5 min. The powder was collected by centrifugation, washed two times with 1,000 µl de-ionized, filtered (12 kDa cut-off) and autoclaved water and resuspended in de-ionized, filtered (12 kDa cut-off) and autoclaved water (975 µl) and 25 µl Protinase K (20 mg/ml, Invitrogen) and transferred to dialysis tubing as described in Melchior et al. (2008) [15].

### Hair

The DNA extraction method used for hair (Egtvedpigen and the individuals from Borum Eshøj) is described in Gilbert et al. (2007) [9] as the “CPH laboratory”-method.

### Haplogroup assignment

Haplogroup (Hg) affiliations were assigned following the established rules and definitions [31]–[33]. Population affinities by haplogroup/type frequencies were determined by comparison with published data for extant populations in Europe and Near East using a private mtDNA database maintained by one of us (TK). To graphically illustrate haplogroup consistency and substitution differences among sequences a most parsimonious tree was chosen from the median joining network [34] relating the HVR-1 sequences and coding region substitutions for 56 ancient individuals from eight different sites.

### Statistical analysis of diversity

Sequence data from nt 16064–16400 obtained from 201 extant ethnic Danes, comprising 193 sequences from the Department of Forensic Genetics, University of Copenhagen and eight sequences from archaeologists and anthropologists collaborating with us on the Galgedil material [16], [35], were compared with sequence data for the same region for individuals from Simonsborg, Bøgebjerggård, Skovgaarde, Galgedil, Kongemarken and Riisby, as these sites each comprise six or more individuals. The diversity within the seven population samples was calculated using the expression:
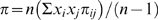
Where π is the nucleotide diversity, *n* the number of sequences, *x_i_* and *x_j_* the frequencies of the *i* ^th^ and *j* ^th^ sequences respectively and π*_ij_* the proportion of different nucleotides between them (see e.g. [36]).

## Results

During this genetic survey of ancient Southern Scandinavians we have attempted to retrieve and sequence authentic mtDNA from 92 individuals from 18 locations ranging from 7,000 YBP to AD 1450. Reproducible results were obtained for 56 individuals from eight of the locations. The results for 37 of these individuals have previously been published; this includes data for the Danish Roman Iron Age (Skovgaarde and Bøgebjerggård [15]), the Viking Age (Galgedil [16]) and the Early Christian Period (Kongemarken [17]). Here we present mtDNA sequences for 19 individuals from the Neolithic site Damsbo, the Early Bronze Age site Bredtoftegård, the Roman Iron Age settlement at Simonsborg and the medieval cemetery at Riisby (Tables 1, 2, 3). DNA extraction and amplification was also attempted from the remains of 10 additional individuals from these sites but no amplifiable DNA or no reproducible results were obtained. Authenticity was considered established when identical results were obtained with extracts from at least two samples from the same individual analysed by two different researchers using different batches of reagents and at a time interval of at least one week. None of the 19 mtDNA sequences matched the mtDNA sequences for any of the staffs (archaeologists, anthropologists and laboratory staff) and unequivocal mtDNA haplogroups were assigned to each of the 19 individuals (Tables 1, 2, 3). Additional data, e.g. the occurrence of the observed ancient haplotype motifs among 15,854 individuals from extant populations of Europe and the Near East, are listed in Table S1.

**Table 1:** Nucleotide substitutions and mtDNA haplogroups assigned for individuals from the Neolithic site Damsbo (4,200 YBP) and the Early Bronze Age site Bredtoftegård (3,300–3,500 YBP).

Individual	Coding sequence	HVR-1 region nt16064–16405	Haplogroup
**D1**	7028T, 12308G	16356C	U4
**D2**	7028T, 12308G	16114A, 16192T, 16256T, 16270T, 16294T	U5a
**Bt1**	7028T, 12308G	16179T, 16356C	U4

D1 and D2, Damsbo; Bt1, Bredtoftegård.

**Table 2:** Nucleotide substitutions and mtDNA haplogroups assigned for individuals from the Roman Iron Age sites Bøgebjerggård (AD 1–400), Simonsborg (AD 1–200) and Skovgaarde (AD 200–400).

Individual	Coding sequence	HVR-1 region nt 16064–16405	Haplogroup
**B1**	7028T, 10034C	16129A, 16223T, 16391A	I
**B2**	7028T	16126C, 16355T, 16362C	R0a
**B3**	7028T, 12308G	16129C, 16183C, 16189C, 16362C	U2e
**B4**	7028C	CRS	H
**B5**	7028T, 10034C	16129A, 16223T, 16304C, 16391A	I
**B6**	7028C	CRS	H
**B7**	7028T, 12308G	16074G, 16189C, 16192T, 16249C, 16270T	U5b
**Si2**	7028C	16189C	H
**Si4**	7028C	16172C, 16311C	H
**Si5**	7028T, 10034C	16129A, 16223T, 16391A	I
**Si6**	7028C	16093C, 16221T	H
**Si8**	7028T, 12308G	16192T, 16270T, 16304C	U5b
**Si9**	7028T, 15607G	16126C, 16294T, 16296T, 16304C, 16362C	T2b
**S1**	7028T, 13708A	16069T, 16126C	J
**S2**	7028T, 12308G	16224C, 16311C	K
**S3**	7028C	16304C	H
**S4**	7028C	16311C	H
**S5**	7028C	16162G, 16266T, 16319A	H
**S6**	7028C	16299G	H
**S7**	7028T, 4580A	16298C	V
**S9**	7028T, 13708A	16069T, 16093C, 16126C	J
**S11**	7028T, 12308G	16093C, 16224C, 16311C	K
**S13**	7028T, 12308G	16343G, 16390A	U3a
**S14**	7028C	16263C, 16319A	H

B1–B7, Bøgebjerggård; Si1–Si9, Simonsborg; S1–S14, Skovgaarde.

**Table 3:** Tabel 3. Nucleotide substitutions and mtDNA haplogroups assigned for individuals from the Viking Age burial sites Galgedil (AD 1000), the Christian cemetery Kongemarken (AD 1000–1250) and the medieval cemetery Riisby (AD 1250–1450).

Individual	Coding sequence	HVR-1 region nt 16064–16405	Haplogroup
**G1**	7028T, 12308G	16126C, 16224C, 16311C, 16320T	K
**G2**	7028C	16278T	H
**G3**	7028C	16093C, 16212G, 16222T, 16255A	H
**G4**	7028C	16213A	H
**G5**	7028T, 12308G	16256T, 16270T, 16399G	U5a
**G6**	7028T, 10034C	16129A, 16223T, 16391A	I
**G7**	7028T, 14470C, 8705C	16189C, 16223T, 16255A, 16278T	X2
**G8**	7028C	16174T	H
**G9**	7028T, 15607G	16126C, 16294T, 16296T, 16304C	T2
**G10**	7028C	16172C, 16304C	H
**G11**	7028T, 12308G	16172C, 16256T, 16399G	U5a
**K1**	7028T, 12308G	16189C, 16318T	U7
**K2**	7028T	16129A, 16223T, 16391A	I
**K3**	7028T	16069T, 16126C	J
**K4**	7028T	16126C, 16174T, 16266T, 16294T, 16304C	T
**K5**	7028C	CRS	H
**K6**	7028C	16221T	H
**K7**	7028T	16129A, 16223T, 16391A	I
**K8**	7028C	16129A, 16316G, 16360T	H
**R1**	7028T, 15607G	16126C, 16153A, 16294T	T2
**R2**	7028T, 12308G	16093C, 16224C, 16311C, 16319A	K
**R3**	7028T, 13708A	16069T, 16126C	J
**R5**	7028C	16261T, 16296T, 16304C	H
**R6**	7028T, 12705T	16147A, 16172C, 16195C, 16223T, 16248T, 16320T, 16355T	N1a
**R9**	7028C	rCRS	H
**R10**	7028T, 13708A	16069T, 16126C	J
**R11**	7028T, 13708A	16069T, 16126C, 16256T	J
**R12**	7028T, 10034C	12129A, 16223T, 16391A	I
**R13**	7028T, 12308G	16189C, 16192T, 16270T, 16398A	U5b

G1–G11, Galgedil; K1–K8, Kongemarken; R1–R13, Riisby.

### Success rate

A marked variability in the success rate was observed among the 18 locations. Table 4 shows additional parameters, such as storage time and handling, which besides the age of the skeletal material may influence the ability to obtain authentic ancient DNA [16], [37], [38]. For the individuals from Galgedil, sampled during excavation, a success rate of 100% was achieved, and for the Neolithic samples, still embedded in soil from Damsbo, a 40% success rate (2/5) was found. In contrast the success rate was nil for several of the highly manipulated skeletal remains (Table 4).

**Table 4:** Success rate, reason for failure, age of site, year of excavation and post-excavation handling for 18 ancient locations.

Site	Success rate	Reason for failure	Age of site	Excavated (year)	Handling
Bøgebakken	0/1	contaminated	7,000 YBP	1975	very handled
Tybrind Vig	0/1	no DNA	6–7,000 YBP	1976	very handled
Hulbjerg	0/5	1, no DNA	4–5,000 YBP	1960–61	handled
		4, contaminated			
Kyndeløse	0/5	3, no DNA	4–5,000 YBP	1937–38	handled
		2, contaminated			
Strø Bjerge	0/2	1, no DNA	4,400 YBP	1978	handled
		1, contaminated			
Damsbo	2/5	no DNA*	4,200 YBP	2006	not handled
Bredtoftegård	1/1		3,300–3,500 YBP	2007	handled
*Borum Eshøj*	0/2	1, no DNA	3,300 YBP	1870	very handled
		1, contaminated			
*Egtvedpigen*	0/1	no DNA	3,300 YBP	1921	very handled
Hestehavebakken	0/1	contaminated	3,100–3,700 YBP	1977	handled
Bøgebjerggård	7/8	contaminated	AD 1–400	1992, 2000	handled
Simonsborg	6/10	contaminated	AD 1–200	1965–1968	handled
Skovgaarde	11/14	contaminated	AD 200–400	1982, 1988	very handled
Himlingeøje	0/1	contaminated	AD 200–400	1940's–1950's	very handled
Varpelev	0/1	no DNA	AD 200–400	1876–1877	very handled
Galgedil	11/11		AD 1000	2005	not handled
Kongemarken	8/10	contaminated	AD 1000–1250	1996–2000	handled
Riisby	10/13	contaminated	AD 1250–1450	1986	handled

The two sites in italic indicate the use of hair as aDNA source.

Success rate is listed as number of individuals where unequivocal assignment of mtDNA haplotypes was possible out of total number of individuals tested. The next column states the reason for failure of the remaining samples. “No DNA” signifies “not sufficient amount of DNA” in the extract for a PCR to be successfully performed.

*The second tooth from one of the three “unsuccessful” individuals from Damsbo did not contain sufficient DNA for a replication to be conducted.

### Kinship

Possible maternal kinship was observed for two males from Bøgebjerggård (B4 and B6, Table 2) who shared the uninformative root of Hg H (rCRS), two females from Kongemarken (K2 and K7, Table 3) who shared the root type of Hg I and a young female and young male from Riisby (R3 and R10, Table 3) who shared the root type of Hg J. No maternal kinship was observed for two individuals from Galgedil (G9 and G11) who were buried in the same grave.

### Phylogeny

**Figure 2:**
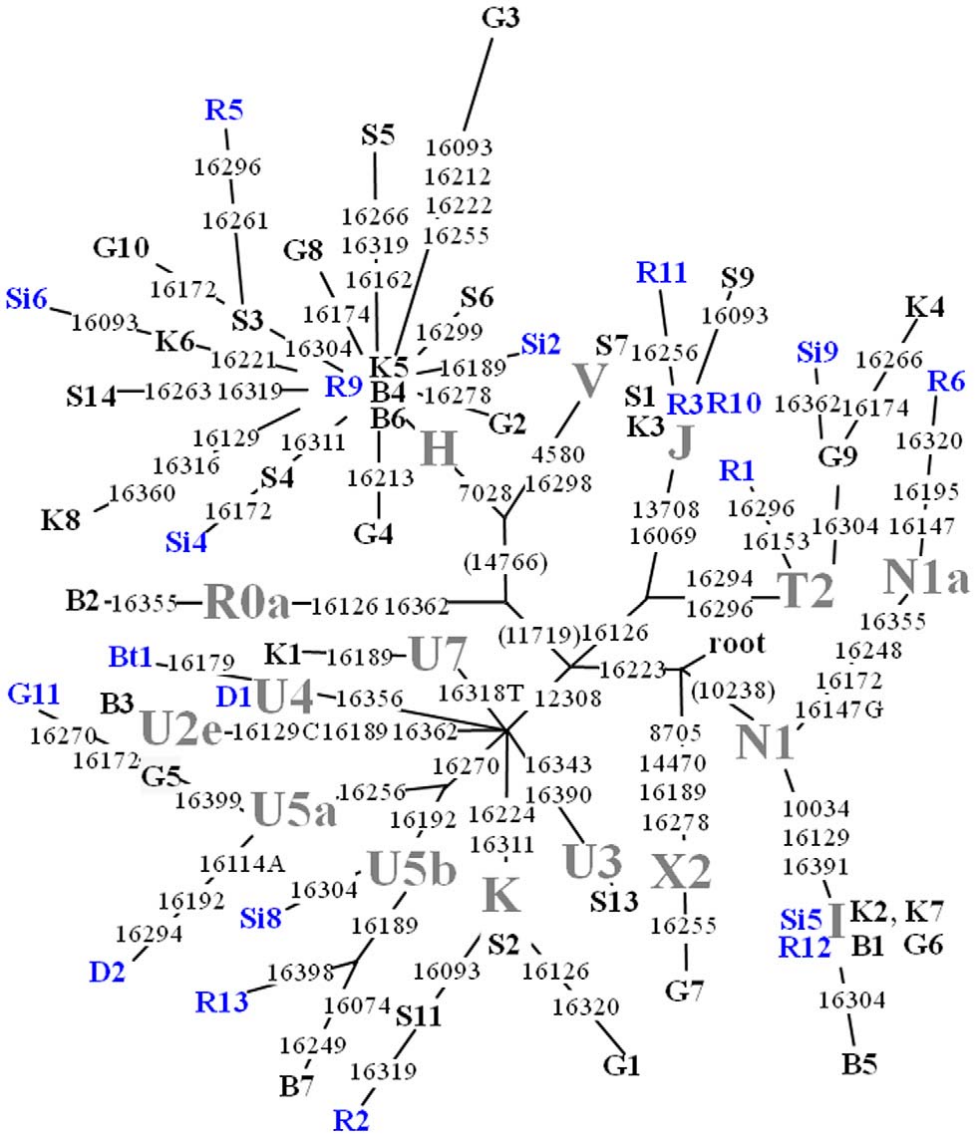
Median joining network of 56 ancient Danes. Median joining network relating the 56 ancient HVR-1 sequences (nt 16064–16405) genotyped for mtDNA haplogroup defining coding region substitutions. The samples are from the Danish Medieval Age: Riisby (R:10 individuals), Danish Viking Age: Galgedil (G:11 individuals) and Kongemarken (K:Eight individuals), Danish Roman Iron Age: Bøgebjerggård (B:Seven individuals), Skovgaarde (S:11 individuals) and Simonsborg (Si:Six individuals), Danish Early Bronze Age Bredtoftegård (Bt: One individual) and Danish Neolithic Age Damsbo (D: Two individuals). Sample codes correspond to Tables 1, 2, 3, haplogroups are shown in grey front and the blue and black sample codes correspond to published data (black) and unpublished data (blue). Variable sites are shown along the branches of the network. Substitutions at nucleotide positions 11719, 14766 and 10238 (shown in parentheses) were inferred from the haplogroup tree drawn using completely sequenced mtDNA genomes [34], [59], [60]. Reticulations between haplogroups, e.g. R0a vs. JT (16126 parallelism) and U1 vs. U7 (16189 parallelism) were solved manually considering phylogenetic analyses based on complete sequence data. L3 is used as the root.

The phylogenetic relationship of the ancient mtDNA sequences was assessed through median-joining network approach using L3 as the outgroup (Fig. 2). We used this approach to 1) test for unusual mutation combinations which would indicate problems such as contamination or the existence of multiple alternative phylogenetic solutions due to extensive homoplasy 2) compare the haplogroup structure of the ancient samples with that of modern day populations. Given the combination of coding region and hypervariable markers such an inference is possible. No unusual substitution motifs indicating odd mosaic sequence combinations were observed nor were unexpected haplogroup compositions at the ancient sites, as for example the high N1a frequency previously observed in LBK sites (*Linearbandkeramik* or linear pottery culture) [10]. The spectrum of haplogroups in our ancient samples is broadly in concordance with the haplogroup variation observed among extant populations in Western Eurasia (H, I, J, K, T, U, V, W, X).

### Unusual or rare Hg's in a South Scandinavian context

Several haplogroups which are rare or absent among the extant population of southern Scandinavia were observed. Hg's R0a and U7 have been discussed previously [15], [17]. Here we note the finding of Hg N1a in the Medieval Riisby (Table 3), which seems to be common among early European LBK farmers [10], a rare Hg T2 motif in the Iron Age settlement Simonsborg (Table 2) and Hg U5a and Hg U4 at the Early Bronze Age site Bredtoftegård and Neolithic Damsbo (Table 1).

### Comparison of haplogroup frequencies

Table 5 shows the occurrence of haplogroups among ancient Danes and Britons and modern Danes and Scandinavians. Using *G*-tests, no significant deviations were observed among the extant populations or between the ancient Britons and the ancient Danes, despite the two ancient population samples show a surplus of Hg T and Hg I, respectively. We have previously observed a high frequency of Hg I's among Iron Age villagers (Bøgebjerggård) and individuals from the early Christian cemetery, Kongemarken [16], [17]. This trend was also found for the additional sites reported here, Simonsborg, Galgedil and Riisby. The overall frequency of Hg I among the individuals from the Iron Age to the Medieval Age is 13% (7/53) compared to 2.5% for modern Danes [35]. The higher frequencies of Hg I can not be ascribed to maternal kinship, since only two individuals share the same common motif (K2 and K7 at Kongemarken). Except for Skovgaarde (no Hg I's observed) frequencies range between 9% and 29% and there seems to be no trend in relation to time. No Hg I's were observed at the Neolithic Damsbo and the Bronze Age site Bredtoftegård, where all three individuals harboured Hg U4 or Hg U5a (Table 1). We tested for homogeneity among the haplotype distribution of the 53 pooled samples of ancient Danes from the Iron Age to the Medieval Age and the extant Danes sample with a *G*-test. The two distributions turned out to be significantly different (*G* = 18.48, df = 9, *p* = 0.03). The difference was mainly caused by an elevated frequency of Hg I among the ancient sample. Likewise the finding of exclusively Hg Us in the Neolithic and Bronze Age samples indicate that these early people differed from later inhabitants. If the Neolithic and Bronze Age populations had the same Hg frequencies as extant Danes, the probability of picking by chance three Hg U individuals among them would be *x* ^3^ = 0.0020, where *x* is the frequency of Hg U among extant Danes (12.5%). Likewise, if they had the same Hg U frequency (*y*, 15.1%) as ancient Danes from AD 1–1450 the probability of observing three Hg U among them would be *y* ^3^ = 0.0034. Both values indicate a significant, increased frequency of Hg U among the Neolithic and Early Bronze Age populations.

**Table 5:** mtDNA haplogroup frequencies among ancient and extant populations.

Hg	Ancient Danes(%)(No)		AncientBritons(%)(No)		ExtantDanes(%)(No)		ExtantScandinavians(%)(No)	
H	35.7	(20)	-	-	41.0	(83)	48.5	(313)
I	12.5	(7)	2.1	(1)	2.5	(5)	1.9	(12)
J	10.7	(6)	8.3	(4)	13.0	(26)	10.2	(66)
K	7.1	(4)	2.1	(1)	9.5	(19)	5.0	(32)
T	7.1	(4)	22.9	(11)	8.5	(17)	8.8	(57)
U	19.6	(11)	16.7	(8)	12.5	(25)	16.3	(105)
V	1.8	(1)	4.2	(2)	3.5	(7)	5.7	(37)
W	0.0	(0)	4.2	(2)	0.5	(1)	1.6	(10)
X	1.8	(1)	4.2	(2)	1.0	(2)	0.6	(4)
Others	3.6	(2)	35.5	(17)	8.0	(16)	1.0	(9)
Total	100	(56)	100	(48)	100	(201)	100	(645)

The haplogroup frequencies of ancient Danes compared with ancient Britons (AD 300–1000) [20], Extant Danes [35] and Extant Scandinavians [61]. In the study by Töpf et al. (2006) [20] the frequency of Hg H was included among Hg “others”.

### Genetic diversity

The genetic diversity was calculated for the locations with six or more successfully typed individuals and was compared to the diversity for 201 modern Danes (Table 6). It is noted that four of the six locations (Bøgebjerggård, Simonsborg, Galgedil and Riisby) showed a considerably higher diversity than present day Danes, while the remaining two sites (Skovgaarde and Kongemarken) had diversities similar to that of modern Danes.

**Table 6:** Nucleotide diversity (π) among six ancient sites compared to extant Danes.

	Individuals	Segregating sites	π
Bøgebjerggård	7	14	0.0147 (±0.0028)
Skovgaarde	11	14	0.0105 (±0.0011)
Simonsborg	6	14	0.0144 (±0.0025)
Kongemarken	9	14	0.0122 (±0.0020)
Galgedil	10	22	0.0154 (±0.0019)
Riisby	9	25	0.0183 (±0.0030)
Extant Danes	201	82	0.0125 (±0.0006)
Sum	253	96	0.0128 (±0.0005)

## Discussion

The present study was conducted under observation of the strict criteria for aDNA work which were formulated over the past decade after it was realized that many early studies were flawed due to undetected contaminating DNA [26]–[29]. Using these criteria we have obtained reproducible, authentic results from human skeletal remains from various sites and various time periods from the Neolithic to the Medieval with an overall success rate of 60% (56/92, see Table 4). However, contamination with extant human DNA proved to be a serious problem. Thus the success rate was nil for several sites with highly manipulated skeletal remains (Table 4). In contrast a 100% success rate was obtained with individuals (Galgedil) that had been untouched for 1,000 years at the time of sampling (no evidence of contamination) and a 2/5 success rate with individuals still embedded in soil from the Neolithic (Damsbo). It was not possible to extract sufficient DNA from two of the three remaining individuals from Damsbo, and sufficient DNA was only recovered from one of two unhandled teeth from a third individual thus preventing replication. Factors such as the age of the remains, the temperature at the archaeological site, the composition of the soil, the time that has elapsed since exhumation and treatment with chemicals to preserve the physical appearance influence the chance of finding surviving DNA (Table 4) [37]–[40]. Contaminating human DNA constitutes a severe problem since it may be indistinguishable from the endogenic DNA (especially if the donors are ethnically related to the subjects), and exogenic DNA may, after relatively few years, accumulate damage patterns that are similar to those of the endogenic DNA [39]. The ratio of contaminating DNA to endogenic DNA may be reduced via thorough treatment with hypochlorite [30], [41]–[43] and/or reduction of the length of the target DNA [44]. The latter is especially promising since the ratio of endogenic to exogenic DNA increases exponentially as the target size is decreased [43]. In a recent study [14] Neolithic samples were sequenced by targeting short fragments (∼80bp) and taking advantage of new high throughput sequencing systems that allow the simultaneous analysis of large numbers of sequences. However, to convincingly rule out the influence of contaminating sequences, it is strongly advised that ancient human material should be sampled at the time of exhumation.

The extent to which early European farmers were immigrants or descendants of resident hunter-gatherers (replacement vs. cultural diffusion) has been widely debated [45]–[48], and new genetic elements have recently been added [8], [10], [14], [19]. A high frequency of Hg U lineages, especially U5, has been inferred for pre-Neolithic Europeans based on modern mtDNA data, with Hg U5 being fairly specific to Europe [46]. In our Neolithic samples from Damsbo (4,200 YBP) and the Bronze Age sample from Bredtoftegård (3,300–3,500 YBP) all three individuals harboured clade U (Table 1), and in concordance with the findings of Bramanti et al. (2009) [8] and Malmström et al. (2009) [14] we identified Hg U4 (Bt1 and D1) and U5a (D2). The presence of solely Hg U4 and U5a in our Neolithic and Early Bronze age samples is noteworthy given that Hg U4 and U5 have frequencies around 1–5% and 5–7%, respectively, among Europeans today [49]. Bramanti et al. (2009) [8] associated these two haplogroups with the Mesolithic populations of Central and Northern Europe while noticing their significant decline among the first farmers of the LBK Culture. The present findings indicate that predominantly haplogroup U lineages persist among Neolithic/Bronze Age population samples in Southern Scandinavia and it may point to regional variation in the penetrance rate of these lineages across cultural shifts in different areas of North Europe. Given our small sample sizes from these crucial time periods further studies are certainly required. However, the frequency of Hg U4 and U5 declines significantly among our more recent Iron Age and Viking Age Danish population samples to the level observed among the extant Danish population. Our study therefore would point to the Early Iron Age and not the Neolithic Funnel Beaker Culture as suggested by Malmström et al. (2009) [14], as the time period when the mtDNA haplogroup frequency pattern, which is characteristic to the presently living population of Southern Scandinavia, emerged and remained by and large unaltered by the subsequent effects of genetic drift. In contrast to Hg U4, which is only found in the Neolithic and Early Bronze Age samples, Hg U5 was observed in ∼9% (5/53) of the remaining ancient samples and identified at all sites except Kongemarken and Skovgaarde.

Several haplogroups which are rare (<0.5% [50]) or absent among the extant population of southern Scandinavia were observed among the 56 ancient individuals. Thus Hg N1a was identified in individual R6 from Riisby. This haplogroup is very rare throughout Europe today (0.2% [49]). It is highlighted here since it was the predominant haplogroup (25%) observed among the 24 individuals from graves with the first European LBK farmers 7,500 YBP [10]. The low frequency of this haplogroup today indicates that the early farmers did not have a strong genetic influence on the extant European female lineages [10] and that farming culture may have spread without the people originally carrying these ideas. Haplogroup T2b was identified in individual Si9 from Simonsborg. This specific haplotype motif is only observed among extant populations in Eastern Europe (0.06%). This finding coincides with previous findings of Eastern European types and haplotype motifs, U7 (K1), H_16174_ (G8) and U5b (R13) in the ancient material [16], [17] and confirms archaeological evidence for contacts between Southern Scandinavia and Eastern Europe [3].

The observation of a high incidence of Hg I's among the ancient Danes (13%, Tables 1, 2, 3) is interesting since this is not reflected in extant population samples (1.9–2.5%, Table 5) and neither is it observed in ancient population samples from Italy, Spain, Great Britain, central European hunter-gatherers, early central European farmers and Neolithic samples (3 out of 184 individuals ∼1.6%) [8], [10], [14], [18]–[21]. Hg I may therefore have had more pronounced differences in frequency among ancient populations and could have been frequent in ancient Southern Scandinavia. The lower frequency observed among extant Danes may be due to later immigration events or genetic drift.

We found only three cases of possible maternal kinship at the eight burial sites. Two males, B4 and B6, from the Iron Age site, Bøgebjerggård (Table 2), and two females, K2 and K7, from the Early Christian cemetery, Kongemarken (Table 3) have been dealt with previously [15], [17]. B4 and B6 may be sharing the frequent Hg H by chance as these two burials differ by 100–200 years (see Table S1). Finally a female and a male, R3 and R10, from the Medieval Riisby share the root of Hg J (Table 3). The organisation of the burials in rows has been proposed to indicate kinship [51], but R3 and R10 were neither buried in the same row nor in the same layer and Hg J is relatively frequent (3%). Our finding therefore suggests that maternal kinship was not frequent in the small ancient societies. This finding is in agreement with the high genetic diversity we observed for these population samples (see below). It should be noted, however, that while a mtDNA haplogroup mismatch excludes maternal relationship a match has limited informative value and given its maternal mode of inheritance mtDNA haplotyping gives no information as to paternal kinship. A profound kinship analysis would require multi-locus analyses such as those performed in paternity testing [52].

The finding that the genetic diversity in four out of six ancient population samples (Bøgebjerggård, Simonsborg, Galgedil and Riisby) is higher than among extant Danes (Table 6) is in agreement with similar findings by Töpf et al. (2007) [53] for ancient Britons. It is noted that the two remaining population samples (Skovgaarde and Kongemarken) did not show a low diversity, just a diversity similar to that in the extant sample. A possible explanation for the lower diversity observed at Skovgaarde could be due to the village social status. Skovgaarde represented the highest level of society and may therefore have consisted of only a few families who intermarried with a limited number of other high-level families in Scandinavia and Northern Europe thereby reducing the diversity [15]. It was suggested that the greater genetic diversity observed for ancient (AD 300–1000) British population samples could reflect the effect of a genetic bottleneck such as the Black Death that raged in Europe in the Medieval Ages [53], killing roughly one third of the European population [54]–[56]. Interestingly, we found the greatest diversity (π = 0.183, see Table 6) in the medieval population sample (Riisby) from this time period AD 1250–1450. However, given the population size at this time (around 1 million in Denmark at AD 1250 [57]) it seems unlikely that the Black Death would significantly reduce the genetic diversity via bottleneck effect. Thus, even an 80% reduction in population size has been shown not to influence the genetic diversity among African buffaloes [58]. Even though dramatic, we find it unlikely that the Black Death is the sole cause of the reduced genetic diversity of mtDNA today. To explore the possible effect of the Black Death on genetic diversity, it would be intriguing to analyse remains from medieval burials sites with well characterised layers from various time periods before and after AD 1350.

## Supporting Information

Table S1 Grave id, sex, age, substitutions in coding sequence and HVR-1 region and assigned haplogroups for all 92 individuals included in the study
(0.15 MB DOC)

Table S2 The archaeological sites and human remains
(0.07 MB DOC)

## References

[1] Jensen J (1995). The Prehistory of Denmark.

[2] Ethelberg P (2000). Skovgaarde. Ein Bestauttungsplatz mit reichen Frauengräbern des 3. Jhs.n.Chr. auf Seeland.

[3] Roesdahl E (1987). The Vikings.

[4] Forster P (2004). Ice Ages and the mitochondrial DNA chronology of human dispersals: a review.. Phil Trans R Soc Lond B.

[5] Forster P, Matsumura S (2005). Evolution. Did early humans go north or south?. Science.

[6] Macaulay V, Hill C, Achilli A, Rengo C, Clarke D (2005). Single, Rapid Coastal Settlement of Asia Revealed by Analysis of Complete Mitochondrial Genomes.. Science.

[7] Thangaraj K, Chaubey G, Kivisild T, Reddy AG, Singh VK (2005). Reconstructing the origin of Andaman Islanders.. Science.

[8] Bramanti B, Thomas MG, Haak W, Unterlaender M, Jores P (2009). Genetic discontinuity between local hunter-gatherers and central Europe's first farmers.. Science.

[9] Gilbert MTP, Djurhuus D, Melchior L, Lynnerup N, Worobey M (2007). mtDNA from hair and nail clarifies the genetic relationship of the 15th century Qilakitsoq Inuit mummies.. Am J Phys Anthropol.

[10] Haak W, Forster P, Bramanti B, Matsumura S, Brandt G (2005). Ancient DNA from the First European Farmers in 7500-Year-Old Neolithic Sites.. Science.

[11] Helgason A, Lalueza-Fox C, Ghosh S, Sigurethardottir S, Sampietro ML (2009). Sequences from first settlers reveal rapid evolution in Icelandic mtDNA pool.. PLoS Genet.

[12] Krause J, Briggs AW, Kircher M, Maricic T, Zwyns N (2010). A Complete mtDNA Genome of an Early Modern Human from Kostenki, Russia.. Curr Biol.

[13] Lamers R, Hayter S, Matheson CD (2009). Postmortem miscoding lesions in sequence analysis of human ancient mitochondrial DNA.. J Mol Evol.

[14] Malmström H, Gilbert MTP, Thomas MG, Brandström M, Stora J (2009). Ancient DNA reveals lack of continuity between neolithic hunter-gatherers and contemporary Scandinavians.. Curr Biol.

[15] Melchior L, Gilbert MTP, Kivisild T, Lynnerup N, Dissing J (2008). Rare mtDNA haplogroups and genetic differences in rich and poor Danish Iron-Age villages.. Am J Phys Anthropol.

[16] Melchior L, Kivisild T, Lynnerup N, Dissing J (2008). Evidence of Authentic DNA from Danish Viking Age Skeletons Untouched by Humans for 1,000 Years.. PLoS ONE.

[17] Rudbeck L, Gilbert MTP, Willerslev E, Hansen AJ, Lynnerup N (2005). mtDNA analysis of human remains from an early Danish Christian cemetery.. Am J Phys Anthropol.

[18] Sampietro ML, Caramelli D, Lao O, Calafell F, Comas D (2005). The Genetics of the Pre-Roman Iberian Peninsula: A mtDNA Study of Ancient Iberians.. Ann Hum Genet.

[19] Sampietro ML, Lao O, Caramelli D, Lari M, Pou R (2007). Palaeogenetic evidence supports a dual model of Neolithic spreading into Europe.. Proc R Soc B.

[20] Töpf AL, Gilbert MTP, Dumbacher JP, Hoelzel AR (2006). Tracing the phylogeography of human populations in Britain based on 4th–11th century mtDNA genotypes.. Mol Biol Evol.

[21] Vernesi C, Caramelli D, Dupanloup I, Bertorelle G, Lari M (2004). The Etruscans: a population-genetic study.. Am J Hum Genet.

[22] Di Benedetto G, Nasidze IS, Stenico M, Nigro L, Krings M (2000). Mitochondrial DNA sequences in prehistoric human remains from the Alps.. Eur J Hum Genet.

[23] Lalueza-Fox C, Sampietro ML, Gilbert MTP, Castri L, Facchini F (2004). Unravelling migrations in the steppe: mitochondrial DNA sequences from ancient Central Asians.. Proc Roy Soc Lond B.

[24] Noonan JP, Coop G, Kudaravalli S, Smith D, Krause J (2006). Sequencing and Analysis of Neanderthal Genomic DNA.. Science.

[25] Römpler H, Rohland N, Lalueza-Fox C, Willerslev E, Kuznetsova T (2006). Nuclear Gene Indicates Coat-Color Polymorphism in Mammoths.. Science.

[26] Cooper A, Poinar HN (2000). Ancient DNA: do it right or not at all.. Science.

[27] Gilbert MTP, Bandelt HJ, Hofreiter M, Barnes I (2005). Assessing ancient DNA studies.. Trend Ecol Evol.

[28] Hofreiter M, Serre D, Poinar HN, Kuch M, Pääbo S (2001). Ancient DNA.. Nat Rev Genet.

[29] Pääbo S, Poinar H, Serre D, Jaenicke-Despres V, Hebler J (2004). Genetic analyses from ancient DNA.. Annu Rev Genet.

[30] Dissing J, Kristinsdottir MA, Friis C (2008). On the elimination of extraneous DNA in fossil human teeth with hypochlorite.. J Archaeol Sci.

[31] Richards MB, Macaulay VA, Bandelt HJ, Sykes BC (1998). Phylogeography of mitochondrial DNA in western Europe.. Ann Hum Genet.

[32] Torroni A, Achilli A, Macaulay V, Richards M, Bandelt HJ (2006). Harvesting the fruit of the human mtDNA tree.. Trend Genet.

[33] van Oven M, Kayser M (2009). Updated comprehensive phylogenetic tree of global human mitochondrial DNA variation.. Hum Mutat.

[34] Bandelt HJ, Forster P, Röhl A (1999). Median-joining networks for inferring intraspecific phylogenies.. Mol Biol Evol.

[35] Mikkelsen M, Sørensen E, Rasmussen EM, Morling N (2009). Mitochondrial DNA HV1 and HV2 variation in Danes.. Forensic Sci Int Genet.

[36] Jobling MA, Hurles ME, Tyler-Smith C (2004). Human Evolutionary Genetics, Origin, Peoples and Disease.

[37] Burger J, Hummel S, Hermann B, Henke W (1999). DNA preservation: a microsatellite-DNA study on ancient skeletal remains.. Electrophoresis.

[38] Pruvost M, Schwarz R, Correia VB, Champlot S, Braguier S (2007). Freshly excavated fossil bones are best for amplification of ancient DNA.. Proc Nat Acad Sci U S A.

[39] Sampietro ML, Gilbert MTP, Lao O, Caramelli D, Lari M (2006). Tracking down Human Contamination in Ancient Human Teeth.. Mol Biol Evol.

[40] Smith CI, Chamberlain AT, Riley MS, Stringer C, Collins MJ (2003). The thermal history of human fossils and the likelihood of successful DNA amplification.. J Hum Evol.

[41] Malmström H, Stora J, Dalen L, Holmlund G, Götherström A (2005). Extensive Human DNA Contamination in Extracts from Ancient Dog Bones and Teeth.. Mol Biol Evol.

[42] Salamon M, Tuross N, Arensburg B, Weiner S (2005). Relatively well preserved DNA is present in the crystal aggregates of fossil bones.. Proc Natl Acad Sci U S A.

[43] Malmström H, Svensson EM, Gilbert MTP, Willerslev E, Götherström A (2007). More on contamination: The use of asymmetric molecular behaviour to identify authentic ancient human DNA.. Mol Biol Evol.

[44] Briggs AW, Good JM, Green RE, Krause J, Maricic T (2009). Targeted retrieval and analysis of five Neandertal mtDNA genomes.. Science.

[45] Ammerman AJ, Cavalli-Sforza LL (1984). The Neolithic Transition and the Genetics of Populations in Europe.

[46] Richards M, Macaulay V, Hickey E, Vega E, Sykes B (2000). Tracing European founder lineages in the near eastern mtDNA pool.. Am J Hum Genet.

[47] Shennan S (2009). Evolutionary demography and the population history of the European early neolithic.. Hum Biol.

[48] Wittle A (1996). Europe in the Neolithic. The creation of New Worlds.

[49] Röhl A, Brinkmann B, Forster L, Forster P (2001). An annotated mtDNA database.. Int J Legal Med.

[50] Coble MD, Just RS, O'Callaghan JE, Letmanyi IH, Peterson CT (2004). Single nucleotide polymorphisms over the entire mtDNA genome that increase the power of forensic testing in Caucasians.. Int J Legal Med.

[51] Kieffer-Olsen J (1993). Grav og gravskik i det middelalderlige Danmark.

[52] Budowle B, van DA (2008). Forensically relevant SNP classes.. Biotechniques.

[53] Töpf AL, Gilbert MTP, Fleischer RC, Hoelzel AR (2007). Ancient human mtDNA genotypes from England reveal lost variation over the last millennium.. Biol Lett.

[54] Harrison D (2000). Stora Döden.

[55] Nordberg M (1987). Den dynamiske middelalder.

[56] Ziegler P (1982). The Black Death.

[57] Liebgott N-K (1998). Danmark i Middelalderen.

[58] Heller R, Lorenzen ED, Okello JB, Masembe C, Siegismund HR (2008). Mid-Holocene decline in African buffalos inferred from Bayesian coalescent-based analyses of microsatellites and mitochondrial DNA.. Mol Ecol.

[59] Kivisild T, Shen PD, Wall DP, Do B, Sung R (2006). The role of selection in the evolution of human mitochondrial genomes.. Genetics.

[60] Loogvali EL, Roostalu U, Malyarchuk BA, Derenko MV, Kivisild T (2004). Disuniting Uniformity: A Pied Cladistic Canvas of mtDNA Haplogroup H in Eurasia.. Mol Biol Evol.

[61] Helgason A, Siguroardottir S, Nicholson J, Sykes B, Hill EW (2000). Estimating Scandinavian and Gaelic ancestry in the male settlers of Iceland.. Am J Hum Genet.

