# Supplementary material for: Genetic Diversity among Ancient Nordic Populations
Source: PLoS One. 2010 Jul 30;5(7):e11898. doi: 10.1371/journal.pone.0011898 (PMC2912848; doi:10.1371/journal.pone.0011898)
Supplement: Table S1 — Grave id, sex, age, substitutions in coding sequence and HVR-1 region and assigned haplogroups for all 92 individuals included in the study (0.15 MB DOC) [file pone.0011898.s001.doc]

**Table S1. Grave id, sex, age, substitutions in coding sequence and HVR-1 region and assigned haplogroups for all 92 individuals included in the study.**

| Individual  (grave number, sex, age (years)) | Coding sequence | HVR-1 region  nt16064 -16405 | Haplogroup | Occurrence among 15,854 individuals from extant populations of Europe and Near East |
| --- | --- | --- | --- | --- |
| **Tybrind Vig** Mesolithic(6-7,000YBP) | | | | |
| **Tv1,** ♀, 15-17 | No results | No results | - |  |
| **Bøgebakken** Mesolithic (7,000YBP) | | | | |
| **Bø1,** H . | No results | No results | - |  |
| **Hulbjerg** Neolithic Passage grave (4-5,000YBP) | | | | |
| **H1,** 666, ♂, 35-45 | No results | No results | - |  |
| **H2,** PB52, ♂, 20-30 | No results | No results | - |  |
| **H3,** 2162-0692 P81, ♂, 25-30 | No results | No results | - |  |
| **H4,** 679, ♀, 20-25 | No results | No results | - |  |
| **H5,** 7162-559, ?? | No results | No results | - |  |
| **Kyndeløse** Neolithic passage grave (4-5,000YBP) | | | | |
| **Ky1,** 338, ♂, 30-50 | No results | No results | - |  |
| **Ky2,** 339, ♂, 20-25 | No results | No results | - |  |
| **Ky3,** 323, ♀, 20-25 | No results | No results | - |  |
| **Ky4,** 344, ♀, 20-25 | No results | No results | - |  |
| **Ky5,** 356, ♂, 20-30 | No results | No results | - |  |
| **Damsbo** Neolithic passage grave (4,200 YBP) | | | | |
| **D1,** 4401x9468,?,? | 7028T, 12308G | 16356C | U4 | Common throughout Europe, 0.8%. |
| **D2,** 4401x9423,?,? | 7028T, 12308G | 16114A, 16192T, 16256T, 16270T, 16294T | U5a | Rare Hg but found throughout Europe, 0.4%. |
| **D3,** 4401x9581,?,? | No results | No results | - |  |
| **D4,** 4401x9668,?,? | No results | No results | - |  |
| **D5,**4401x3527(x10597),?,? | No results | No results | - |  |
| **Strø Bjerge** Neolithic cist grave (4,400 YBP) | | | | |
| **Sb1,** 1, ?, ? | No results | No results | - |  |
| **Sb2,** II, ?, ? | No results | No results | - |  |
| **Bredtoftegård** Neolithic cist grave (4,000 YBP) | | | | |
| **Bt1,** AS157/2007, ♂ 35-40 | 7028T | 16179T, 16356C | U4 | Common throughout Europe, 0.8%. |
| **Hestehavebakken** Early bronze Age (3,100-3,700 YBP) | | | | |
| **Hh1,** 5, ♂, 60 | No results | No results | - |  |
| **Egtvedpigen** Early Bronze Age (3,300 YBP) | | | | |
| **E,** ♀, 16-18 | No results | No results | - |  |
| **Borum Eshøj** Early Bronze Age (3,300 YBP) | | | | |
| **Be1,** ♂, 20-22 | No results | No results | - |  |
| **Be2,** ♂, 50-60 | No results | No results | - |  |
| **Bøgebjerggård** Roman Iron Age (AD 1-400) | | | | |
| **B1,** E**,** ♀, 35-45 | 7028T, 10034C | 16129A, 16223T, 16391A | I | Rare Hg but spread throughout Europe, 0.2%. |
| **B2,** 4, ♂, 30-40 | 7028T | 16126C, 16355T, 16362C | R0a | Only observed in South-Eastern Europe. Frequent among Bedouins in the Arabian Peninsula [9]. |
| **B3,** 6, ♂, 40 | 7028T, 12308G | 16129C, 16183C, 16189C, 16362C | U2e | Rare everywhere only observed in one individual in England. |
| **B4,** 7**,** ♂, 25-30 | 7028C | CRS | H | Common throughout Europe, 12.2%. |
| **B5,** B, ♀, 25-30 | 7028T, 10034C | 16129A, 16223T, 16304C, 16391A | I | Rare North European type, 0.07%. |
| **B6,** A, ♂, 18-19 | 7028C | CRS | H | Common throughout Europe, 12.2%. |
| **B7,** marts 2000, ♀, 35-45 | 7028T, 12308G | 16074G, 16189C, 16192T, 16249C, 16270T | U5b | Not observed in extant humans (yet), but related subtypes in Norwegians and Scots. |
| **B8,** A4, ?, ? | No results | No results | - |  |
| **Simonsborg** Roman Iron Age (AD 1-200) | | | | |
| **Si1,** 10, ♂, 45-60 | No results | No results | - |  |
| **Si2,** 21, ♀, 35-55 | 7028C | 16189C | H | Common Hg throughout Europe, 1.1%. |
| **Si3,** 5, ♀, 35-55 | No results | No results | - |  |
| **Si4,** 6, ♂, 25-40 | 7028C | 16172C, 16311C | H | Rare Hg, 0.03%. |
| **Si5,** 7, ♀, 20-35 | 7028T, 10034C | 16129A, 16223T, 16391A | I | Rare Hg but widely spread throughout Europe, 0.2%. |
| **Si6,** 9, ♂, 20-35 | 7028C | 16093C, 16221T | H | Rare Hg but found throughout Western Europe, 0.15%. |
| **Si7,** 11, ♂, 35-55 | No results | No results | - |  |
| **Si8,** 14, ♀, 20-35 | 7028T, 12308G | 16192T, 16270T, 16304C | U5b | Rare Hg but widely spread throughout Europe, 0.06% |
| **Si9,** 18, ♀, 35-55 | 7028T, 15607G | 16126C, 16294T, 16296T, 16304C, 16362C | T2b | Rare Hg observed in Eastern Europe, 0.07% |
| **Si10,** 22, ♂, 35-55 | No results | No results | - |  |
| **Skovgaarde** Roman Iron Age (AD 1-200) | | | | |
| **S1,** 207, ?, 14-16 | 7028T, 13708A | 16069T, 16126C | J | Common Hg widely spread throughout Europe, 3.0%. |
| **S2,** 5, ♂, 50 | 7028T, 12308G | 16224C, 16311C | K | Common throughout Europe |
| **S3,** 4, ♀, 40-50 | 7028C | 16304C | H | Common throughout Europe |
| **S4,** 208, ?, 14-16 | 7028C | 16311C | H | Common throughout Europe |
| **S5,** 7, ♀, 30-35 | 7028C | 16162G, 16266T, 16319A | H | Not observed in extant humans (yet), but related subtypes in one Norwegians, one Swede, one Russian and one Estonian. |
| **S6,** 1943, ♀, 25-30 | 7028C | 16299G | H | Rare type observed mainly in Northern-Europe, 0.15% |
| **S7,** 209, ♀, 20 | 7028T, 4580A | 16298C | V | Common throughout Europe. |
| **S8,** 400, ♀, 25-30 | No results | No results | - |  |
| **S9,** 204, ♀, 55 | 7028T, 13708A | 16069T, 16093C, 16126C | J | Rare throughout Europe, 0.05%. |
| **S10,** 202, ♂?, 18-20 | No results | No results | - |  |
| **S11,** 205, ♀?, 25 | 7028T, 12308G | 16093C, 16224C, 16311C, | K | Common throughout Europe. |
| **S12,** 9, ♀, 55-58 | No results | No results | - |  |
| **S13,** 8, ♀, 20-30 | 7028T, 12308G | 16343G, 16390A | U3a | Common throughout Europe. |
| **S14,** 210, ♀, 45 | 7028C | 16263C, 16319A | H | Rare type, only observed in one individual in Southern Germany. |
| **Himlingøje** Late Roman Iron Age (AD 200-400) | | | | |
| **Hi1,** 1, ♂, adult | No results | No results | - |  |
| **Varpelev** Late Roman Iron Age (AD 200-400) | | | | |
| **V1,** 1, ♂, adult. | No results | No results | - |  |
| **Galgedil** Viking Age (AD 700-1000) | | | | |
| **G1,** AMA, ♀, 50+ | 7028T, 12308G | 16126C, 16224C, 16311C, 16320T | K | No exact match neither in European database nor in haplogroup K database [10], related haplotypes exist with either 16126 or 16320 transition. |
| **G2,** ALZ, ♂, 45+ | 7028C | 16278T | H | Rare but widely spread haplotype throughout Europe, 0.16%. |
| **G3,** AKJ, ♀?, 40-50 | 7028C | 16093C, 16212G, 16222T, 16255A | H | No exact match in the database. Related haplotypes found in Croatian Island population and a Cornish sample. |
| **G4,** ANO, ♂?, 35-45 | 7028C | 16213A | H | Rare but widely spread haplotype throughout Europe, 0.05%. |
| **G5,** ALX, ♂?, 30-40 | 7028T, 12308G | 16256T, 16270T, 16399G | U5a | Common haplotype throughout Europe, 0.92%. |
| **G6,** AXE, ♂, 50+ | 10034C | 16129A, 16223T, 16391A | I | Rare Hg but spread throughout Europe, 0.2%. |
| **G7,** AJG, ♀, 20-30 | 7028T, 14470C, 8705C | 16189C, 16223T, 16255A, 16278T | X2 | Rare but widely spread haplotype throughout Europe, 0.11%. |
| **G8,** BFQ, ♀, 50+ | 7028C | 16174T | H | Rare type observed in Eastern Europe, 0.02%. |
| **G9,** AQQ(1572), ♂, 25-35 | 7028T, 15607G | 16126C, 16294T, 16296T, 16304C | T2 | Common haplotype throughout Europe, 1.71%. |
| **G10,** AQP, ♀, 45+ | 7028C | 16172C, 16304C | H | Rare but widely spread haplotype throughout Europe, 0.03%. |
| **G11,** AQQ(1573), ♂, 25-30 | 7028T, 12308G | 16172C, 16256T, 16399G | U5a | No exact match, but related haplotype 172-256-270-(399), and its derived variants infreq. observed in Sweden, Hungary, Sicily Scotland, (4/15354). |
| **Kongemarken** Viking Age (AD 1000-1250) | | | | |
| **K1,** A396, ♂, 45+ | 7028T, 12308G | 16189C, 16318T | U7 | Not present among Europeans, but common in India and among western Siberian tribes. |
| **K2,** A367, ♀, 25-30 | 7028T | 16129A, 16223T, 16391A | I | Rare Hg but spread throughout Europe, 0.2%. |
| **K3,** A368, ♀, 45+ | 7028T | 16069T, 16126C | J | Common Hg widely spread throughout Europe, 3.0%. |
| **K4,** A362, ♀, 45+ | 7028T | 16126C, 16174T, 16266T, 16294T, 16304C | T | No exact match in the database. Related haplotypes found throughout Europe. |
| **K5,** A211, ♀, 30-40 | 7028C | CRS | H | Common throughout Europe, 12.2%. |
| **K6,** A386, ♂, 25-40 | 7028C | 16221T | H | Rare but found throughout Europe, 0.12%. |
| **K7,** A363, ♀, 18 | 7028T | 16129A, 16223T, 16391A | I | Rare Hg but spread throughout Europe, 0.2%. |
| **K8,** A395 ,♀?, 15 | 7028C | 16129A, 16316G, 16360T | H | Not exact match in European database nor in haplogroup H database, related types exist with either 16129/16316, 16316 or 360. |
| **K9,** A269**,** ♀, ? | No results | No results | - |  |
| **K10**, A260, ♂, ? | No results | No results | - |  |
| **Riisby** Medival Age (AD 1150-1500) | | | | |
| **R1,** 1, ♀, 20 | 7028T, 15607G | 16126C, 16153A, 16294T | T2 | Rare Hg but found primarily in Scandinavia, 0.1% |
| **R2,** 5, ♂, 40+ | 7028T, 12308G | 16093C, 16224C, 16311C, 16319A | K | Rare Hg, 0.04%. So far only found in Scotland (5 ind.) and Northern Germany (1 ind.) |
| **R3,** 6, ♀, 15-16 | 7028T, 13708A | 16069T, 16126C | J | Common Hg widely spread throughout Europe, 3.0%. |
| **R4,** 14, ♂, 30-40 | No results | No results | - |  |
| **R5,** 10, ♂, 35-40 | 7028C | 16261T, 16296T, 16304C | H | No exact match in European database nor in Hg H database, related types exist with either 16261/16304 (France, Bosnia, Ukraine, Russia) or 16296. |
| **R6,** 24, ♀, 17 | 7028T, 12705T | 16147A, 16172C, 16195C, 16223T, 16248T, 16320T, 16355T | N1a | Very rare Hg, 0.006%. So far only identified in one individual from Northern Germany. |
| **R7,** 23, ♂, 20-25 | No results | No results | - |  |
| **R8,** 21, ♂, 50+ | No results | No results | - |  |
| **R9,** 16, ♂, 30-40 | 7028C | CRS | H | Common throughout Europe, 12.2%. |
| **R10,** 18, ♂, 15-17 | 7028T, 13708A | 16069T, 16126C | J | Common throughout Europe, 3.0%. |
| **R11,** 62, ♂, 40+ | 7028T, 13708A | 16069T, 16126C, 16256T | J | Rare Hg, 0.04%, so far only found in Northern Europe in 2 Scott’s, 2 Norwegians, 1 German and 1 Icelander. |
| **R12,** 47, ♂, 18-19 | 7028T, 10034C | 12129A, 16223T, 16391A | I | Rare Hg but spread throughout Europe, 0.2%. |
| **R13,** 61, ♂, 40+ | 7028T, 12308G | 16189C, 16192T, 16270T, 16398A | U5b | Rare Hg observed in N/E-Europe, 0.04%. |

The last column compares the specific haplotype motif observed with 15,854 entries in a private database maintained by one of us (TK). Haplotypes occurring at >0.5% in the database are considered as “common”, following the frequency criteria used in Coble et al. (2004) study [11]. It should be noted that rare haplogroups that occur across wide geographic space might occur due to parallel mutations in the HVR-1 region.
