# Supplementary material for: Genetic Diversity among Ancient Nordic Populations
Source: PLoS One. 2010 Jul 30;5(7):e11898. doi: 10.1371/journal.pone.0011898 (PMC2912848; doi:10.1371/journal.pone.0011898)
Supplement: Table S2 — The archaeological sites and human remains (0.07 MB DOC) [file pone.0011898.s002.doc]

**Table S2. The archaeological sites and human remains**

**Tybrind Vig**

(401521-6, FMH 2033, AS 009/dk).

Tybrind Vig is a Mesolithic settlement located on the Western side of Funen, and is dated to approximately 6-7,000 YBP. It was originally sitting on the bank of the cove but was later flooded. An exposed grave from the early period of the settlement was discovered in 1976 by sports divers. The grave contained a 15-17 year old female with a child skeleton (1-3 months old) located on the chest. The female and child was exhumed in 1980 during an underwater excavation. An attempt to obtain DNA from a tooth from the young female was made, due to the possibly preserving effect of the calcareous and oxygen deficient mud surrounding the skeletons on the sea bed. Due to the uniqueness of the material only one tooth was used and it did not yield any DNA.

Bøgebakken (Henriksholms-Bøgebakken)

(Søllerød Parish, Sokkelund District, København County. Sb157, NMI 1047/75, AS 3/75).

Bøgebakken is located in North Zealand and is one of the oldest Mesolithic burial sites in Scandinavia from around 7,000 YBP. The site was excavated in 1975 and comprises 17 graves containing a total of 22 well-preserved skeletons. A single individual has been analysed so far to assess the possibility of obtaining DNA from this site. The results are not consistent between the two analysed teeth, and the possibility of obtaining consistent results from other individuals is therefore not promising.

Hulbjerg

(Magleby Parish, Langelands Sønder District, Svendborg County. Sb72, NM 655/62, AS 7/62).

Hulbjerg is a Neolithic passage grave located on the southern tip of the island of Langeland. The burial mound seems to have been used between 4-5,000 YBP. The presence of four burial layers suggests that the mound has been used for several centuries. The passage grave was excavated in 1960-61 and contained at least 36 adults and 17 children [1]. The floor of the grave consists of a spongy organic layer. Analysis of five individuals was carried out, but none of the individuals showed consistent results. In addition several of the results did not make phylogenetic sense.

Kyndeløse

(Kirke Hyllinge Parish, Voldborg District, København County. Sb 6, NM 205/38).

Kyndeløse is a double chamber Neolithic passage grave located in North-West Zealand. The burial mound seems to have been used from 4-5,000 YBP. The burials appear to have comprised around 50 individuals in each chamber, but due to the bones being piled together as new burials were added to the mound, the exact number is unclear. The chambers were excavated in 1937-1938. The floor of the grave consists of a spongy organic layer as was also seen in Hulbjerg. Analysis of five individuals was carried out, but as for Hulbjerg no consistent results were obtained, and the haplotypes did not make phylogenetic sense.

**Damsbo (Damsbo Mark)**

(Jordløse Parish, Sallinge District, Svendborg County. Sb41, FHM 4401, AS 83/2009).

Damsbo is a Neolithic passage grave located in South-West Funen. The grave was partly destroyed but burial layers could easily be detected when the grave was excavated in 2006. The grave was constructed approximately 5,200 YBP, but the burial layers are 1,000 years younger indicating the grave may have been cleared and reused. The grave comprised more than 1,000 human bones. During excavation in 2006 five mandibles were recovered and send to the Research Laboratory still embedded in soil. Teeth from the five individuals were removed taking care to prevent contamination with modern human DNA (described in details in [2]).

Strø Bjerge

(Strø Parish, Strø District, Frederiksborg County. Sb 9, NM 2221/77, AS 6/95).

Strø Bjerge is a destroyed Neolithic cist grave located in North Zealand. The grave was used 3,700-4,400 YBP. Excavation of the grave was carried out in 1978, and 201 human bone fragments, originating from at least 7 individuals were identified. Analyses were carried out on two individuals, but no consistent results were obtained, and the “possible” haplotypes did not make phylogenetic sense.

**Bredtoftegård**

(Lille Lyngby Parish, Strø District, Frederiksborg County. Sb 59, NFH A0380, AS 157/2007).

Bredtoftegård is located in North Zealand and is an Early Bronze Age (~3,300-3,500 YBP) cist grave containing the skeleton of a 35-40 years old male. Excavation was carried out in the summer of 2007, and teeth for aDNA analysis of the male were obtained in the autumn of 2007. Bredtoftegård represented a unique opportunity to include freshly excavated Early Bronze Age material, even though the site only comprised one individual.

**Hestehavebakken**

(Kalundborg Parish, Ars District, Holbæk County. Sb11).

Hestehavebakken is located in West Zealand. Excavation was carried out in 1977, and several burned bones and a fragmented skeleton of an approximately 60 year old woman was exhumed. The skeleton is dated to the Early Bronze Age, (3,100-3,700 YBP). Three teeth from the female skeleton were analysed, but no consistency between the results was obtained.

**Bøgebjerggård**

(Røstofte, Øster Egesborg Parish, Bårse District, Storstrøms County. Sb132, SMV 7273, AS 41/02).

Further details have previously been published [3].

**Skovgaarde**

(Udby Parish, Bårse District, Præstø County. Sb47, SMV 7021, AS 44/89).

Further details have previously been published [3].

**Simonsborg**

(Alsted Parish, Alsted District, Sorø County. Sb 36, NM 558/65 & 586/66, AS 4/67& 12/68).

Simonsborg is located in Central Zealand and is from the Early Roman Iron Age (AD 1-200). Simonsborg is one of the largest and best preserved burial sites from this period in Denmark. Excavation of the site was carried out from 1965 to 1968. The graves were all simple inhumations with little or no grave goods and believed to be a burial ground for the common people [4]. Ten individuals were analysed from this site.

Himlingøje

(Himlingøje Parish, Bjæverskov District, Præstø County. Sb16, NM 1200/75, AS 13/78).

Himlingøje is located in East Zealand. The inhumations are from the Late Roman Iron Age, AD 200-400. Himlingøje was excavated in the 1940’s, 1950’s and during the years 1977-1985. The oldest find though, emerged as early as in 1928. The majority of the burials emerged during gravel digging and was therefore not recorded archaeologically. The site consists of 13 richly equipped burials, but the most of the skeletal material is very poorly preserved. The burials represent an aristocratic family with wide connections to similar families in Scandinavia, the European continent and in the Roman Empire [1,5]. Analysis of one individual was carried out, but no consistent results were obtained. Due to the very poor preservation of the material further analyses were abandoned.

Varpelev (Varpelev Vest)

(Varpelev Parish, Stevns District, Præstø County. Sb6, NMI 19674-85).

Varpelev is located in East Zealand just 3 km from Himlingøje. The inhumations are from the Late Roman Iron Age, AD 200-400 [1]. Varpelev is thought to have been a political and social centre connected to Himlingøje. The site was excavated in two parts. The first was excavated in 1861 and contained a single richly furnished grave. The second part was constructed in a gravel bank, and the first graves were discovered during gravel digging in 1876. Further investigations took place in 1876-1877. This site comprises 10-15 inhumations, two of them richly furnished. As seen for Himlingøje the material from Varpelev was poorly preserved and analysis of one of the individuals showed no consistent result, and further analyses were abandoned.

**Galgedil**

(Otterup Parish, Lunde District, Odense County. Sb8, OBM 4520, AS 04/01).

Galgedil is a non-Christian Viking Age burial site located in the Northern part of Funen. The analysis of ten individuals from Galgedil has previously been published [2], but subsequently an additional individual (G11) was analysed and included in the study. G11 was buried in a double grave with G9 but with G9 placed on a terrace 30 cm higher than G11. DNA analysis was carried out to detect a possible maternal kinship.

**Kongemarken**

(Sankt Jørgensbjerg Parish, Sømme District, Københavns County. Sb132, ROM 1810, AS 3/10).

Details have previously been described [6].

**Riisby**

(Bårse Parish, Bårse District, Præstø County. Sb2, NM 1073, AS 14/86).

Riisby is located in Southern Zealand. The site is an ordinary parish churchyard from the Medieval Ages, and seems to have functioned from AD 1150 to around AD 1500. Excavation of the site was carried out in 1986, and 68 inhumations in up to five layers were uncovered. Two of the graves, R2 (grave 5) and R4 (grave 14), showed some remarkable grave goods. One man had a pilgrim’s shell (in Danish “Ibsskal”) acquired on pilgrimage to Santiago de Compostela, Spain on his hand and the remains of a possible pilgrim’s staff and an amulet on his chest. The other grave contained two pilgrim’s shells on the individuals’ chest [7]. Thirteen individuals were analysed from this site, among them the two males, R2 and R4. The ten individuals, from whom DNA was obtained, could be further subdivided based on the positioning of their arms in the grave. R2, R6, R11 and R12 were estimated to have been buried AD 1250-1350 and R1, R3, R5, R10 and R13 around AD 1350-1450. R9 could not be dated based on arm position [7].

**Hair material included**

Cremation was the predominant burial rite in Denmark during the Bronze Age and up to the Early Roman Iron Age, limiting the material available prior to AD 1. Based on the unsuccessful retrieval of aDNA from the Bronze Age remains from Hestehavebakken, hair samples were included due to experiences with hair samples as good sources of mtDNA [8]. Hair samples from the famous Egtvedpige and two individuals from Borum Eshøj, exhibited on the Danish National Museum, were tested.

Egtvedpigen

(Egtved Parish, Jerlev District, Vejle County. Sb 109, NM B11849, AS 1/81).

The female from Egtved was 16-18 years old when she died 3,300 YBP, and she was buried together with the burned bones of a 5-6 years old child in an oak coffin. She was discovered in 1921, and has due to her uniquely well preserved clothes been studied intensively [1].

Borum Eshøj

(Borum Parish, Framlev District, Århus County. Sb12, NM I 699 & 1396a, AS 35/44).

The two men from Borum Eshøj were buried 3,300 YBP in a burial mound in oak coffins. The skeletal remains, clothing and coffins were exceptionally well preserved. The oak coffins were discovered in 1870 [1].

References for supporting information

1. Jensen J (2006) Danmarks Oldtid 1-4. Copenhagen: Gyldendal.

2. Melchior L, Kivisild T, Lynnerup N, Dissing J (2008) Evidence of Authentic DNA from Danish Viking Age Skeletons Untouched by Humans for 1,000 Years. PLoS ONE 3: e2214.

3. Melchior L, Gilbert MTP, Kivisild T, Lynnerup N, Dissing J (2008) Rare mtDNA haplogroups and genetic differences in rich and poor Danish Iron-Age villages. Am J Phys Anthropol 135: 206-215.

4. Liversage D (1980) Material and Interpretation. The archaeology of Sjælland in the Early Roman Iron Age. Copenhagen: Publications of the National Museum.

5. Lund Hansen U, et alii (Ed) (1995) Himlingøje-Seeland-Europa. Ein Gräberfelt der jüngeren römischen Kaizerzeit auf Seeland, seine Bedeutung und internationalen Beziehungen. Copenhagen: Det Kongelige Nordiske Oldskriftselskab.

6. Rudbeck L, Gilbert MTP, Willerslev E, Hansen AJ, Lynnerup N et al. (2005) mtDNA analysis of human remains from an early Danish Christian cemetery. Am J Phys Anthropol 128: 424-429.

7. Kieffer-Olsen J (1993) Grav og gravskik i det middelalderlige Danmark. Aarhus: The University of Aarhus.

8. Gilbert MTP, Djurhuus D, Melchior L, Lynnerup N, Worobey M et al. (2007) mtDNA from hair and nail clarifies the genetic relationship of the 15th century Qilakitsoq Inuit mummies. Am J Phys Anthropol 133: 847-853.

9. Richards M, Rengo C, Cruciani F, Gratrix F, Wilson JF et al. (2003) Extensive female-mediated gene flow from sub-Saharan Africa into near eastern Arab populations. Am J Hum Genet 72: 1058-1064.

10. Behar DM, Metspalu E, Kivisild T, Achilli A, Hadid Yet al. (2006) The matrilineal ancestry of Ashkenazi Jewry: portrait of a recent founder event. Am J Hum Genet 78: 487-497.

11. Coble MD, Just RS, O'Callaghan JE, Letmanyi IH, Peterson CT et al. (2004) Single nucleotide polymorphisms over the entire mtDNA genome that increase the power of forensic testing in Caucasians. Int J Legal Med 118: 137-146.
